# Machine Learning-Based Hearing Aid Fitting Personalization Using Clinical Fitting Data

**DOI:** 10.1155/2022/1667672

**Published:** 2022-10-15

**Authors:** S. I. M. M. Raton Mondol, Hyun Ji Kim, Kyu Sung Kim, Sangmin Lee

**Affiliations:** ^1^Department of Electrical and Computer Engineering, Inha University, Incheon 22212, Republic of Korea; ^2^Department of Otolaryngology, Inha University Hospital, Incheon 22332, Republic of Korea

## Abstract

The initial software fitting prescribed by the fitting formula largely depends on the patient's hearing loss, which may not be the optimal preference for a particular user. Certain criteria must also be readjusted by an audiologist to meet the user-specific requirements. Therefore, this study focuses on the novel application of a neural network (NN) technique to build a suitable fitting algorithm with prescribed hearing loss and the corresponding preferred gain to minimize the gap between optimized fittings. The algorithm intended to learn the hearing preferences of an individual user such that the initial fitting may be optimized. These findings demonstrate the efficiency of the algorithm, with and without additional features. Using the clinical fitting data, the average mean square error (MSE) for the simple NN algorithm was 5.4183%. By adding additional features to the data, the algorithm performed better, and the average MSE was as low as 5.2530%. However, the algorithm outperformed Company A fitting software, as the MSE was the highest at 5.4748%. As the company's automatic fitting has a noticeable discrepancy with clinical fitting records, the impeccable results from this study can lead to a better path towards fitting satisfaction, thus benefiting the hearing-impaired community to a larger extent.

## 1. Introduction

For patients with hearing loss, good hearing aid fitting is more than just choosing an effective hearing aid (HA) device. Hearing aid fitting formulas calculate the amount of electroacoustic amplification required for people with hearing loss [1]. Modern hearing aids generally follow two steps for fitting. The first step is initial fitting, which is provided by a fitting formula that depends on the patient's hearing loss across frequencies [2, 3]. The second step is fine-tuning. An audiologist requires a final adjustment or correction to fit the user's unique hearing, cognitive characteristics, and personal preferences [4]. Although fine-tuning is crucial to the successful outcome of hearing aid usage, precise initial fitting is a prerequisite for efficient fine-tuning [2–4].

The National Acoustic Laboratories' (NAL) nonlinear (NL) fitting procedure, NAL-NL1 [5, 6], NAL-NL2 [2, 7], and Desired Sensation Level (DSL5.0) [3] are some widely used nonlinear hearing aid fitting formulas to prescribe hearing aid gain. The aim of both NAL-NL1 and NAL-NL2 is to achieve a comfortable level of loudness while optimizing speech intelligibility. However, the theoretical derivation differs in two key points: (i) intelligibility model and (ii) gain constraint. Therefore, NAL-NL2 prescribes more gains at high and low frequencies than at midfrequencies. Furthermore, NAL-NL2 considers the age, sex, experience of using hearing aids, language, and compressor speed of patients [7]. DSL fitting [3], on the other hand, aims to improve speech recognition output in each frequency region by aiming at comfortable listening levels. This procedure uses the desired sensation levels to measure the empirical real-ear-aided gain [3]. Although these fitting formulas differ in their underlying principles, restoring the individual's audibility is the main concern for all of them, which is primarily based on the English language [8].

It has been acknowledged that up to 50% of hearing aid users prefer amplification or compression settings different from those recommended by the manufacturer [9–11]. This is because hearing perception and acoustic settings vary from user to user, and various publications in the literature have examined the fact of self-adjustment or self-tuning of hearing aid fitting compared to one-size-fits-all prescription fitting [12–15]. According to Convery et al. [15], hearing aid users prefer gain settings that differ from those of NAL prescriptions, both under calm and noisy conditions. Additionally, Sandlin et al. [1] examined the advantages of hearing aid personalization by considering different facts in a smartphone-based system.

The dynamic range (DR), the long-term average speech spectrum (LTASS), and the band importance function (BIF) of speech are the most substantial features of language in terms of acoustics and linguistic attributes [8]. Therefore, these characteristics should be considered to restore audibility in individuals with hearing loss by measuring the degree of amplification required across the frequency bands [16]. However, these characteristics differ between Korean and English speech. For example, Noh and Lee [17] reported that the Korean speech showed a different LTASS than the English language. Specifically, the high frequency energy for the Korean LTASS was less than that for the English LTASS. In addition, the DR of Korean speech differs from that of English speech. Jin et al. [18] reported that the DR for Korean speech is narrower than that for English speech at low frequencies. The band importance function represents the relative contribution of speech intelligibility to different frequencies and varies from language to language [19]. The results of previous studies indicate that the Korean language-specific HA fitting formula may provide better audibility for Korean people with hearing loss than formulas based on other languages such as English.

Only a few studies have reported strategies for self-adjustment or self-tuning compression in HA fitting [20–22]. However, none of these studies considered the preferred fitting record from an audiologist for self-fitting. In one of our previous research projects [23], we evaluated the performance of a machine learning-based hearing aid fitting algorithm. We used data extracted from two popular nonlinear hearing aid fitting software programs (NAL-NL1 and NAL-NL2) and trained the model to predict hearing aid fitting prescriptions. The results were satisfactory for an ideal training and testing dataset. Applying a similar approach to a small amount of clinical data from an audiologist could be more challenging and realistic.

Therefore, the purpose of this study was to develop a personalized hearing aid fitting method for Korean users and to compare the differences in gain preferences between the initial fitting of HA devices. We used fitting data for the products of a well-known hearing aid company, which accounts for more than 20% of the global market and distributes its hearing aids through hospitals in Korea. In this study, the hearing aid company will be referred to as Company A. First, the preferred gain for the HA fitting is compared with the initial fitting of Company A. Second, an NN-TL approach is used to predict the preferred HA fitting of hearing loss patients with and without additional features to learn if additional features such as age, sex, or ear type help to obtain better results. In general, this study will provide information on whether HA fitting formulas need to be specific to the native language of the HA user, and additional features will help the algorithm learn better.

The remainder of this paper is organized as follows: Section 2 covers the materials and methods used in this study.  Section 3 discusses the methodology of the study. The experimental results are presented in Section 4, followed by the discussion and conclusions in Sections 5 and 6, respectively.

## 2. Materials and Methods

Many variables related to hearing aids and patient conditions affect hearing aid outcomes. The current study considered only some specific conditions, including patients with sensorineural hearing loss, user experience in HA, behind-the-ear (BTE) HA, 2 cc couplers, and 6 channels when insertion gains were determined for each frequency. The rationale of fitting was to make loudness comfortable while providing sufficient audibility for low (50 dB sound pressure level (SPL)), medium (65 dB SPL), and high (80 dB SPL) sounds.

Clinical fitting data from 978 Korean patients (466 men and 512 women) with mild-to-severe sensorineural hearing loss were used in this study. Data were collected from the Department of Otolaryngology at Inha University Hospital, and no personal information regarding the participants was disclosed.

**Figure 1:**
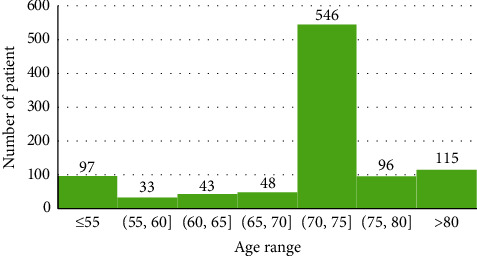
Fitting data distribution based on the patient's age.

The ages of the patients ranged from 1 to 96 years, and the distribution is shown in Figure 1. The age distribution is not uniform as more than half of the patients were between 71 and 75 years.

**Figure 2:**
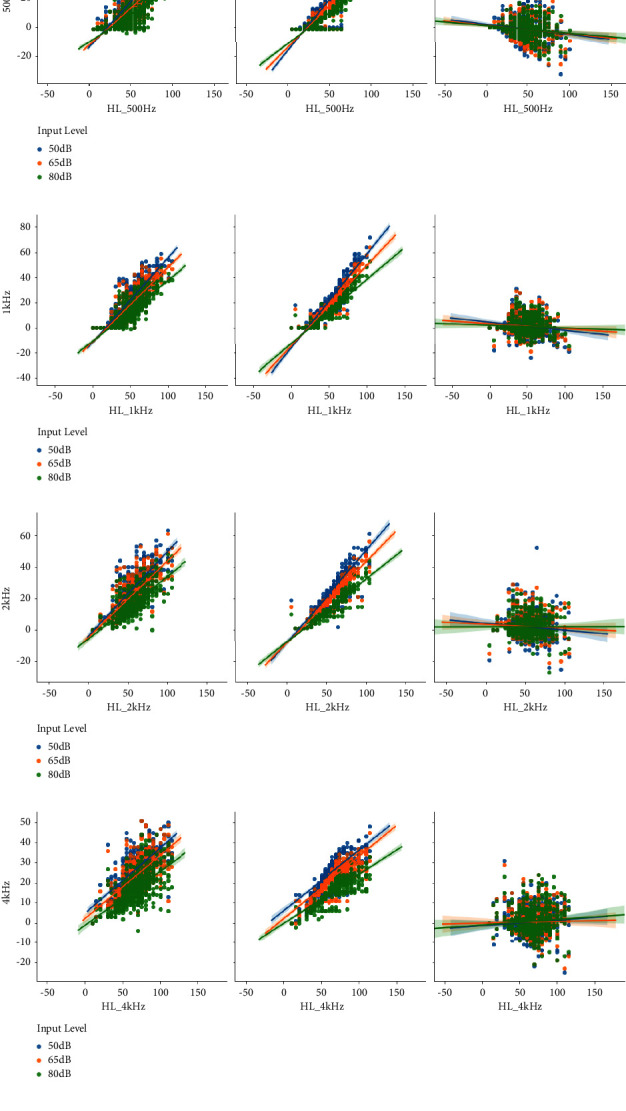
Patient's fitting preferences, initial fitting, and their differences across different input levels and frequency bands.(a) Frequency band 250 Hz. (b) Frequency band 500 Hz. (c) Frequency band 1 KHz. (d) Frequency band 2 KHz. (e) Frequency band 4 KHz. (f) Frequency band 8 KHz.

Figure 2 represents the distribution of fitting preference, initial fitting, and their differences across the 3 input sound levels (as in legend) and 8 frequency bands (as in subfigures a to f). The figure shows that the distribution of the preferred gains is more scattered than that of the initial fitting. In addition, the initial fitting is narrower in range than the preferred fitting.

## 3. Methodology

**Figure 3:**
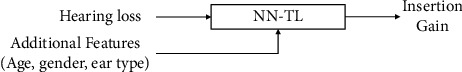
A block diagram of the proposed model.

A simple block diagram of the proposed hearing aid fitting model is shown in Figure 3.

Typically, fitting formulas calculate the insertion gain based on the user's hearing loss information. Here, we added some additional features as shown in the block diagram to feed the neural network model to compute the personalized insertion gain.

**Figure 4:**
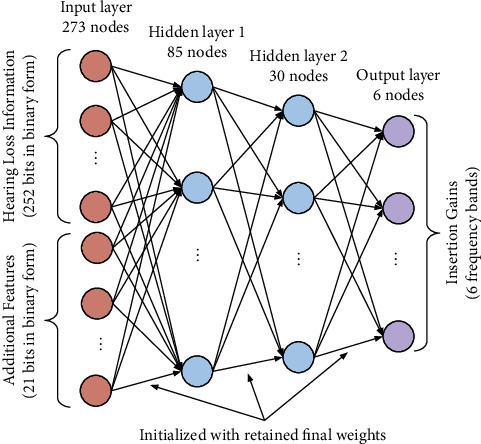
4-layer perceptron neural network.

A preliminary study on a machine learning approach to hearing aid fitting prescriptions was conducted using data from fitting software (NAL-NL1 and NAL-NL2) [23], and the identical model is also adopted here. A neural network model with refined hyperparameters learns the representation of data at multiple levels of abstraction, and the transfer learning approach accelerates the training process and enhances the performance of the model, even in a smaller dataset condition. However, it is important to consider real-time hearing aid fitting data collected from healthcare professionals for Korean patients with hearing loss to obtain a more realistic impression of the algorithm's performance. Additional features, such as age, sex, and ear type, were also considered to determine whether performance was further improved. The architecture implemented here is similar to that of the multilayer perceptron neural network model (MLP-NN) [13], which is based on a feed-forward neural network [24, 25]. In our approach, we only considered the simple supervised training with a refined hyperparameter. The 4-layer perceptron neural network consists of 273 units of the input layer, 85 units of the first hidden layer, 30 units of the second hidden layer, and 6 units of the output layer and is shown in Figure 4.

Instead of random weight initialization, we retained the final weights of the NN model from our previous research [23] and used them here as the initial weights of the transfer learning model. We also considered the exponential weight decay concept for our approach. After initializing all parameters in the training session, feed-forward propagation is calculated first, backpropagation is calculated second, then the parameter is updated, and the loss function is calculated at the end. We use sigmoid, the most classical activation function in all the 4 layers, and stochastic gradient descent (SGD) as an optimizer. The list of hyperparameters (learning rate, batch size, number of epochs, etc.) used throughout the simulations is listed in Table 1.

This experiment uses 900 clinical data for training and 78 clinical data for testing purposes. The training dataset and the test dataset were split with a ratio of 23 : 2. The datasets were selected randomly in such a way that no data appear more than once in the combined training and test sets. The training data are fed into the model in small batches. The ML approach uses training data to construct the mapping function between the result variable and the predictors, while testing data are used to evaluate the trained model's ability to predict the outcome variable.

**Figure 5:**
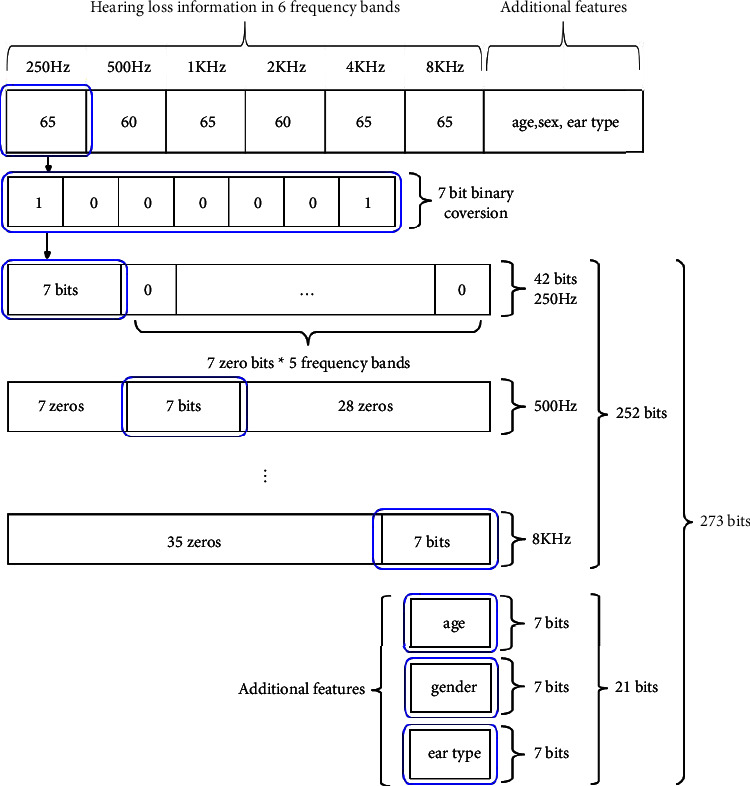
Binary conversion of input features.

The dataset contained information on hearing loss at 6 different frequencies for each participant. In addition to hearing loss and insertion gain information for the corresponding loss, the dataset also contained additional features, such as age, gender, and ear type. We performed a binary conversion of hearing loss data and additional features as preprocessing and then positioned them based on the frequency hierarchy to create more features ((6 frequencies and additional features)*∗*7 bit binary conversion*∗*6 positions) as shown in Figure 5.

## 4. Experimental Results

**Figure 6:**
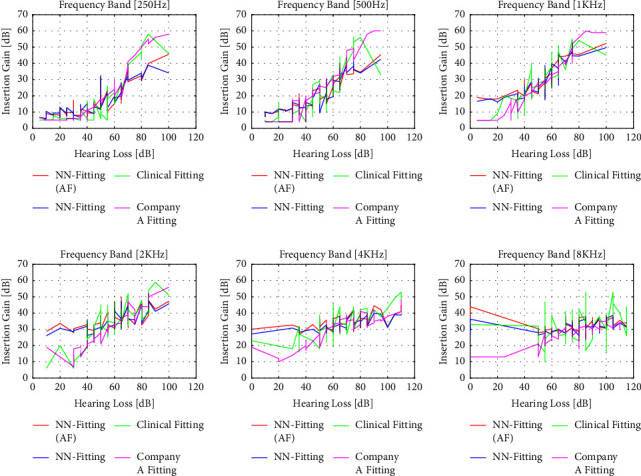
Performance comparison for NN fitting (with and without additional features) vs. clinical fitting and Company A fitting record for the 50 dB input level. Comparisons are made for 250 Hz, 500 Hz, 1 KHz, 2 KHz, 4 KHz, and 8 KHz frequencies.

**Figure 7:**
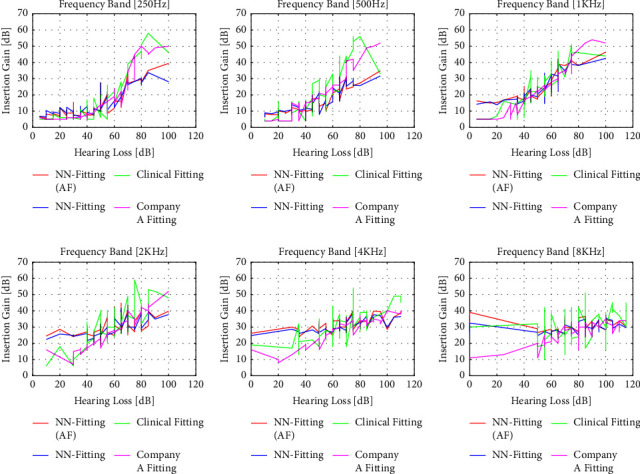
Performance comparison for NN fitting (with and without additional features) vs. clinical fitting and Company A fitting record for the 65 dB input level. Comparisons are made for 250 Hz, 500 Hz, 1 KHz, 2 KHz, 4 KHz, and 8 KHz frequencies.

**Figure 8:**
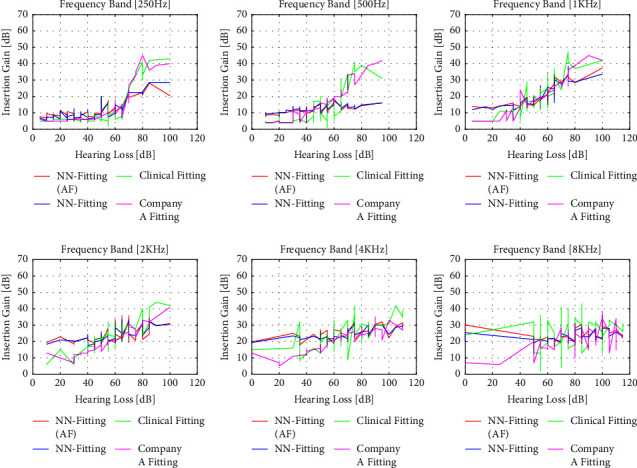
Performance comparison for NN fitting (with and without additional features) vs. clinical fitting and Company A fitting record for the 80 dB input level. Comparisons are made for 250 Hz, 500 Hz, 1 KHz, 2 KHz, 4 KHz, and 8 KHz frequencies.

The performance of the algorithm for 3 different input levels (50 dB, 65 dB, and 80 dB) for initial fitting by Company A, clinical fitting, and NN fitting with and without additional features is shown in Figures 6–8. The magenta line represents the insertion gain extracted from Company A fitting software, the green line corresponds to the clinical insertion gain, and the red and blue lines illustrate the gains predicted by the NN algorithms with and without additional features, respectively. The acronyms used in the performance comparisons have been defined in the legends of the figures. Even though the proposed NN fitting approach followed the trend of the fitting formula well compared to the state-of-the-art approach, it is clear that the NN fitting approach with additional features outperformed the former and that predictions were close to the original fitting records. However, some unexpected gain fluctuations are noticed in some cases but can be neglected

## 5. Discussion

To evaluate the algorithm's performance of predicting fitting records, we employed the mean square error, a commonly used error measure in the literature. A lower value is preferred for the measure, indicating a lower prediction error. The predicted MSE values are listed in Table 2. In the table, the prediction error rates (in percentage) of our proposed NN-TL method for the three different input levels are presented. The results were obtained after the third epoch for each case. From the table, it is clear that the algorithm exhibited slightly better results for each input level when additional features were introduced.

The average MSE for the algorithm was 5.4183%, whereas using additional features, it was 5.2530%. However, the proposed algorithm outperformed the Company A fitting record. It was noticed that the company's automatic fitting has a big difference with real clinical fitting. Although the difference in MSE does not seem great, considering data discrepancy between the groups, the result is impeccable and may have a big effect on hearing aid systems. Moreover, this smaller contribution could show a better path for big companies, thus benefiting the hearing-impaired community to a larger extent. Software fitting is closer to ideal situations where very small or no difference is expected.

Fortunately, all sophisticated modern fitting techniques provide hearing-impaired ears with at least some corrective gain. The ideal fitting approach, which has yet to be devised, would aim to restore all dynamic acoustic characteristics lost due to cochlear and conductive reasons. On the other hand, each patient is treated as a separate experiment with varying and unpredictable results. However, to develop a more precious algorithm, more clinical fitting data should be collected. Overcoming privacy concerns and different approval for data is tedious.

Consequently, the insertion gain computed using the fitting formulas may not be the best gain for patients with hearing loss, and there is no possible baseline for a reasonable prediction error. The desired gain may differ significantly from one subject to another, from male to female, from young to elderly, or from one geographical location to another. Furthermore, we do not know how each fitting program calculates the insertion gain for corresponding hearing loss information. Therefore, this remains a black box.

The use of linguistic characteristics in HA fitting is not a new concept. The tonal language was taken into consideration, and low-frequency gains were boosted when NAL-NL2 was created [2]. This is because pitch changes are more significant for tone perception in tonal languages (e.g., Cantonese and Mandarin) compared to English [2, 26]. However, in contrast to English and tonal languages, the Korean language has distinct qualities. The Korean language possesses unique acoustic properties for LTASS, dynamic range of speech, and BIF as described previously. Compared to tonal languages, such as Mandarin, the Korean language also has different acoustic characteristics such as BIF and DR of speech [18, 19]. Based on these linguistic variances, it appears that the existing HA fitting formulas do not adequately capture the acoustic features of the Korean language.

## 6. Conclusions

We attempted to bridge the gap between fitting satisfaction and hearing loss patient satisfaction by combining this technique with clinical data from an audiologist. Due to regulations and privacy concerns, collecting significant amounts of hearing aid fitting data from audiologists is a tedious task. Therefore, we considered the parameter transfer learning approach so that we could deal with the problem of smaller datasets. The prediction error for the insertion gain is basically the calculated difference between the predictive gain and the initial gain from the fitting formula. Therefore, a very small difference or no difference is expected under ideal conditions. We developed a fitting algorithm using the machine algorithm, and it is better than the company's fitting algorithm. However, to develop a more precious algorithm, more clinical fitting data should be gathered and the characteristics of the components (mic and rec) of the hearing aid should also be considered.

The current study showed both limitations and possibilities for the effects of a language-specific HA fitting formula. In this study, the NN fitting model with improved hyperparameters enabled computational prototypes with many processing layers to learn data representation at several levels of abstraction, while the TL method accelerated training and improved model performance even with a smaller dataset. However, combining hearing aid fitting data collected from healthcare professionals with additional information, such as the patient's own choice of hearing aid type, age, and sex, provides a more realistic impression about the performance of the proposed algorithm. Both the simulation and experimental results demonstrated the effectiveness of the algorithm in achieving preferred hearing outcomes.

## Figures and Tables

**Table 1:** The list of hyperparameters used by the ML algorithm.

Hyperparameters	Value
Activation function	Sigmoid
Optimizer	SGD
Weight penalty	1 *e* − 3
Learning rate	1 *e* − 4
Batch size	100
Number of epochs	3
Number of layers	4
Input layer	273 nodes
Hidden layer 1	85 nodes
Hidden layer 2	30 nodes
Output layer	6 nodes

**Table 2:** MSE comparison for NN-TL and NN-TL_AF.

Input levels	Clinical fitting data		Company A fitting data
	NN-TL (MSE in %)	NN-TL_AF (MSE in %)	NN-TL (MSE in %)
50 dB	5.1120	5.0065	5.1084
65 dB	5.1176	4.9677	5.1180
80 dB	6.0255	5.7850	6.1980
Average	5.4183	5.2530	5.4748

## Data Availability

The simulation experiment data used to support the findings of this study are available from the corresponding author upon request.
